# Phosphatidylinositol 3-Kinase Couples Localised Calcium Influx to Activation of Akt in Central Nerve Terminals

**DOI:** 10.1007/s11064-015-1663-5

**Published:** 2015-07-22

**Authors:** Jessica C. Nicholson-Fish, Michael A. Cousin, Karen J. Smillie

**Affiliations:** Centre for Integrative Physiology, University of Edinburgh, Hugh Robson Building, George Square, Edinburgh, EH8 9XD Scotland, UK

**Keywords:** Endocytosis, Vesicle, Presynapse, Calcium, Exocytosis, Akt, GSK3

## Abstract

The efficient retrieval of synaptic vesicle membrane and cargo in central nerve terminals is dependent on the efficient recruitment of a series of endocytosis modes by different patterns of neuronal activity. During intense neuronal activity the dominant endocytosis mode is activity-dependent endocytosis (ADBE). Triggering of ADBE is linked to calcineurin-mediated dynamin I dephosphorylation since the same stimulation intensities trigger both. Dynamin I dephosphorylation is maximised by a simultaneous inhibition of its kinase glycogen synthase kinase 3 (GSK3) by the protein kinase Akt, however it is unknown how increased neuronal activity is transduced into Akt activation. To address this question we determined how the activity-dependent increases in intracellular free calcium ([Ca^2+^]_i_) control activation of Akt. This was achieved using either trains of high frequency action potentials to evoke localised [Ca^2+^]_i_ increases at active zones, or a calcium ionophore to raise [Ca^2+^]_i_ uniformly across the nerve terminal. Through the use of either non-specific calcium channel antagonists or intracellular calcium chelators we found that Akt phosphorylation (and subsequent GSK3 phosphorylation) was dependent on localised [Ca^2+^]_i_ increases at the active zone. In an attempt to determine mechanism, we antagonised either phosphatidylinositol 3-kinase (PI3K) or calmodulin. Activity-dependent phosphorylation of both Akt and GSK3 was arrested on inhibition of PI3K, but not calmodulin. Thus localised calcium influx in central nerve terminals activates PI3K via an unknown calcium sensor to trigger the activity-dependent phosphorylation of Akt and GSK3.

## Introduction

Central nerve terminals rely on the efficient recycling of a limited supply of synaptic vesicles (SVs) in order to maintain neurotransmission. This is achieved via recruitment of a number of SV endocytosis modes [1, 2]. Clathrin-mediated endocytosis is dominant during low frequency stimulation and generates SVs directly from the plasma membrane whereas ultrafast endocytosis can be triggered by single action potentials at physiological temperatures and generates SVs via an endosomal intermediate [3–5]. During higher stimulation intensities an additional endocytosis mode is triggered to increase retrieval capacity called activity-dependent bulk endocytosis (ADBE). ADBE generates large endosomes direct from the plasma membrane. These bulk endosomes then donate SVs back to the recycling pool via clathrin-dependent mechanisms [2, 6].

Triggering of ADBE is dependent on the dephosphorylation of the large GTPase dynamin I at two serine residues (Ser774 and Ser778) [7]. This event is mediated by the calcium-dependent protein phosphatase calcineurin and only occurs during intense activity [7]. ADBE is maintained across multiple trains of stimuli via the rephosphorylation of dynamin I by cyclin-dependent kinase 5 on Ser778 and then subsequently by glycogen synthase kinase 3 (GSK3) on Ser774 [8].

GSK3 has a high basal activity which is regulated via its phosphorylation status [9]. Phosphorylation of Ser21/9 of GSK3β/α by upstream cascades inhibits this protein kinase activity. We have shown that GSK3 is phosphorylated in central nerve terminals only during high levels of neuronal activity, coincident with dynamin I dephosphorylation [10]. We also identified that the activity-dependent GSK3 kinase was Akt (also known as protein kinase B). This activity-dependent inhibitory signalling cascade ensures that Ser774 on dynamin I is maximally dephosphorylated during periods of intense activity [10]. Importantly, either expression of constitutively active Akt or addition of signalling molecules such as brain-derived neurotrophic factor (BDNF, which activates Akt) reduces ADBE in cultured neurons [10, 11] suggesting that this activity-dependent signalling cascade is essential for efficient presynaptic function during intense neuronal activity.

It is currently unclear how action potential stimulation is transduced into activation of Akt. To address this we monitored the phosphorylation status of both Akt and GSK3 after elevating intracellular free calcium ([Ca^2+^]_i_) via either action potentials or a calcium ionophore. We found that localised [Ca^2+^]_i_ increases via voltage gated calcium channels were essential for Akt activation. This activation was dependent on phosphatidylinositol 3-kinase (PI3K) but not calmodulin activity. This reveals a key role for PI3K in the activity-dependent control of GSK3 activity, but leaves the identity of the calcium sensor still undetermined.

## Materials and Methods

### Materials

Foetal bovine serum was from Biosera. Penicillin/streptomycin and Minimum Essential Medium were from Invitrogen. The primary antibodies phospho-Akt Ser473 and GSK3α/β Ser21/9 were from Cell Signalling. Bovine serum albumin was from Roche. BAPTA-AM, CNQX and AP-5 were from Tocris. EGTA-AM was from Cambridge Bioscience and ionomycin from Merck Chemicals. Secondary antibodies, β-Actin antibody, LY294002, wortmannin and all other chemicals were bought from Sigma.

### Cerebellar Granule Neuron Cultures

Cerebellar granule neuron (CGN) cultures were prepared from the cerebella from P7 Sprague–Dawley rat pups of either sex [12]. Cultures were used between 8 and 14 days in vitro (DIV).

### CGN Lysis Experiments

Cerebellar granule neurons (CGNs) were repolarised in incubation medium (170 mM NaCl, 3.5 mM KCl, 400 µM KH_2_PO_4_, 20 mM TES [*N*-tris(hydroxy-methyl)-methyl-2-aminoethane-sulfonic acid], 5 mM NaHCO_3_, 5 mM glucose, 1.2 mM Na_2_SO_4_, 1.2 mM MgCl_2_, 1.3 mM CaCl_2_ at pH 7.4) for 10 min. This medium was then replaced by media supplemented with (2*R*)-amino-5-phosphonovaleric acid (AP-5, 50 µM), 6-cyano-7-nitroquinoxaline-2,3-dione (CNQX, 10 µM) and various antagonists as described in the respective figure legends. For electrical field stimulation experiments coverslips were placed in a Warner imaging chamber (RC-21 BRFS) and either challenged with a train of 800 action potentials delivered at 80 Hz or no stimulus (basal). In experiments using ionomycin, cells were treated in an identical manner, with the exception that the challenge with 2.5 µM ionomycin or a vehicle control (DMSO) was for 1 min. In all experiments antagonists were present during stimulation with the exception of those using either BAPTA-AM or EGTA-AM. Low calcium incubation medium had the following alterations, CaCl_2_ was reduced to 100 µM, MgCl_2_ was increased to 10 mM and EGTA was added at 50 µM. After stimulation with either action potentials or ionomycin, CGNs were immediately lysed in 25 µl of SDS sample buffer (67 mM Tris, 9.3 % glycerol, 2 mM EGTA, 67 mM SDS, bromophenol blue, 12 % β-mercaptoethanol) and boiled at 95 °C for at least 5 min.

### Western Blotting

Cerebellar granule neuron (CGN) lysates were subjected to Western blotting as described before [12]. Primary antibodies were all used at 1:1000, apart from HRP-coupled Anti-β-Actin (1:50,000). Secondary antibodies were used at 1:5000 except for Anti-β-Actin where no secondary was required. The intensity of the detected chemiluminescence signal was determined using Image J (NIH). Raw densitometry values were normalised firstly to the highest value within an individual blot and then to individual corresponding loading controls (β-Actin, which was also normalised to the highest value). All Western blot data is displayed after normalisation to the basal signal from each blot. All data was analysed using GraphPad Prism and with students t tests performed between basal and stimulated states as well as between stimulated states across conditions.

## Results

### An Intracellular Free Calcium Increase is Essential for Phosphorylation of Akt/GSK3

**Fig. 1:**
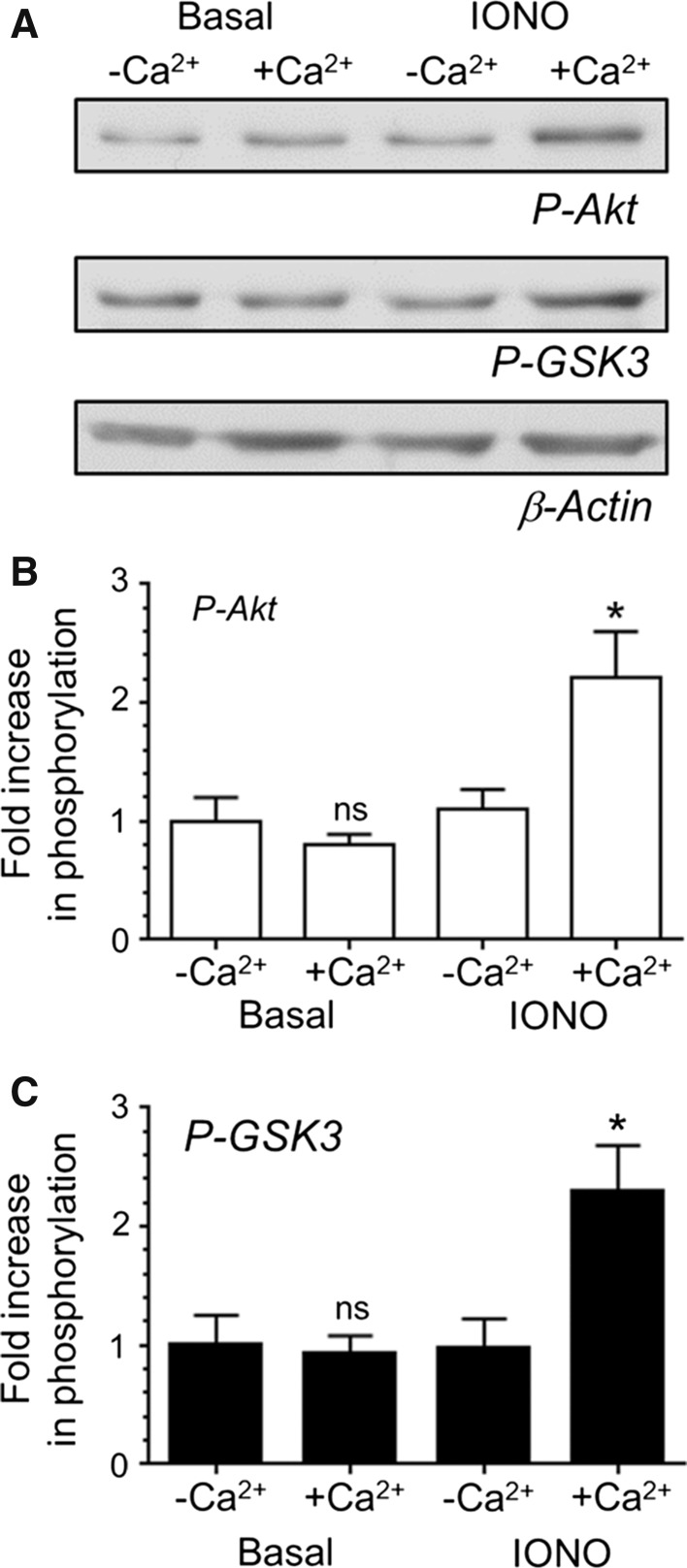
[Ca^2+^]_i_ increases are essential for Akt/GSK3 phosphorylation. CGNs were removed from culture medium and repolarised in incubation medium for 10 min. Cultures were then either rested (Basal) or stimulated with ionomycin (IONO, 5 µM) for 1 min in incubation buffer containing either 1.3 mM (+Ca^2+^) or low (−Ca^2+^) calcium. **a** Representative *blots* are displayed showing either Akt Ser473 phosphorylation (*P*-*Akt*), GSK3α/β Ser21/9 phosphorylation (*P*-*GSK3*) or β-Actin levels (*β*-*Actin*). **b**, **c** The fold increase in phosphorylation of either Akt Ser473 (**b**, *open bars*) or GSK3α/β Ser21/9 (**c**, *closed bars*) is displayed after correction for protein levels using β-Actin and normalisation to the low calcium control. All *error bars* represent ±SEM; n = 5 for P-Akt and n = 4 for P-GSK3 (students *t* test, *ns* non-significant, *p < 0.05, −Ca^2+^ to +Ca^2+^ per condition)

The strict activity-dependence of both Akt and GSK3 phosphorylation [10] suggests an essential requirement for increased [Ca^2+^]_i_. To assess the importance of increased [Ca^2+^]_i_ on both Akt and GSK3 phosphorylation, the calcium ionophore ionomycin was employed to uniformly increase [Ca^2+^]_i_ independent of depolarisation. Phosphorylation of both Akt and GSK3 was observed when CGNs were challenged with ionomycin in the presence of extracellular calcium (Fig. 1). However no evoked increase was observed when this experiment was repeated in low calcium buffer (Fig. 1), indicating that increased [Ca^2+^]_i_ in the absence of membrane depolarization is sufficient to cause activation of this signalling cascade.

**Fig. 2:**
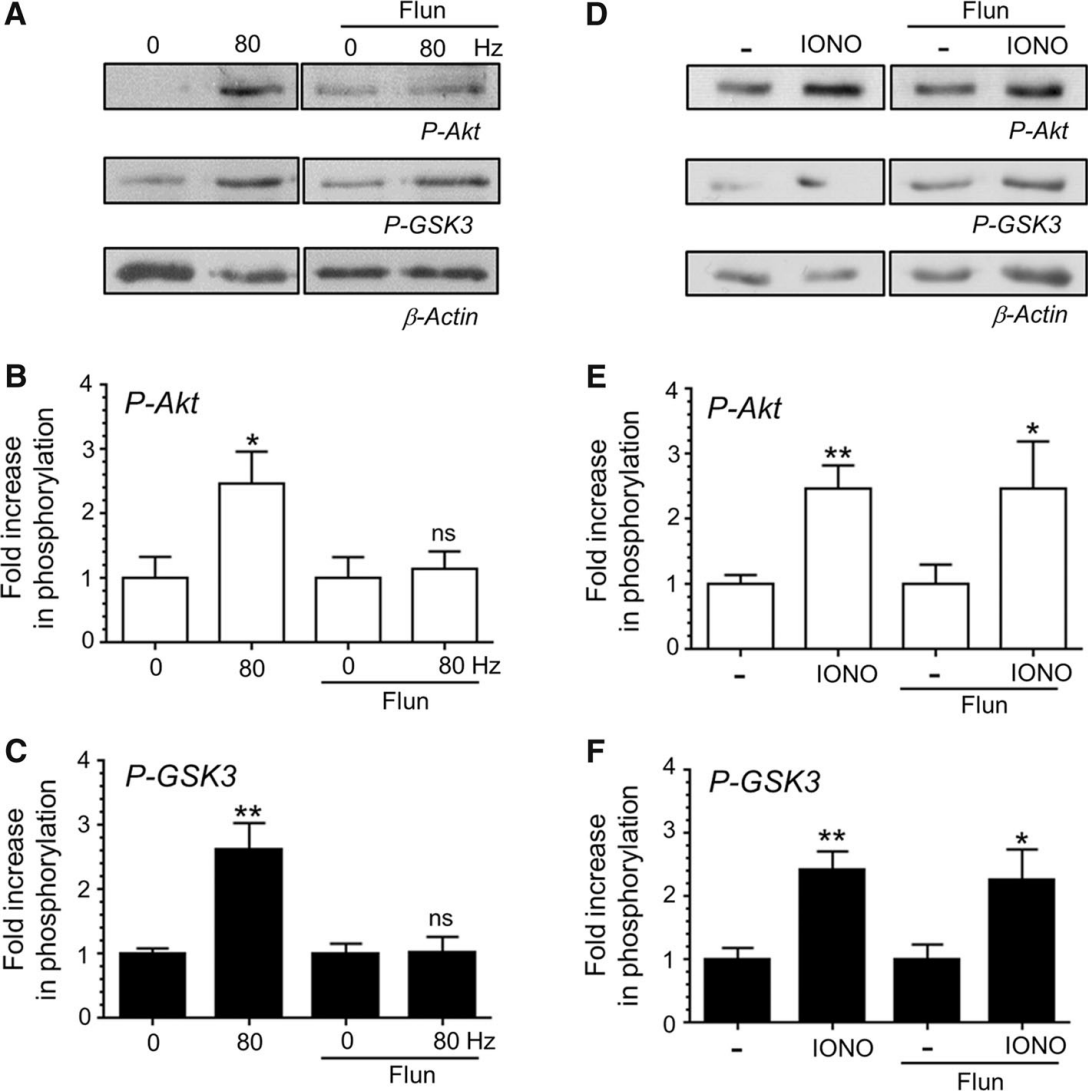
Ca^2+^ influx via voltage-gated calcium channels is essential for Akt/GSK3 phosphorylation. CGNs were removed from culture medium and repolarised in incubation medium for 10 min. Cultures were then incubated with or without incubation medium supplemented with 10 μM flunarizine (Flun) for 10 min. After this point CGNs were left to rest or challenged with either 800 action potentials (80 Hz) or ionomycin (IONO, 2.5 µM for 1 min). **a**, **d** Representative *blots* are displayed showing either Akt Ser473 phosphorylation (*P*-*Akt*), GSK3α/β Ser21/9 phosphorylation (*P*-*GSK3*) or β-Actin levels (*β*-*Actin*) after either action potential (**a**) or ionomycin (**d**) stimulation. **b**, **e** The fold increase in phosphorylation of Akt Ser473 (*open bars*) in response to either action potentials (**b**) or ionomycin (**e**) is displayed. **c**, **f** The fold increase in phosphorylation of GSK3α/β Ser21/9 (*closed bars*) in response to either action potentials (**c**) or ionomycin (**f**) is displayed. In all cases phosphorylation levels were corrected for protein levels using β-Actin and normalisation to the basal controls. All *error bars* represent ±SEM; n = 5 for both P-Akt and P-GSK3 with 80 Hz, n = 6 for P-Akt IONO and n = 7 for P-GSK3 IONO (students *t* test, *ns* non-significant, *p < 0.05; **p < 0.01 either basal to 80 Hz or basal to ionomycin per condition)

Increased presynaptic [Ca^2+^]_i_ during action potential stimulation is usually a result of activation of voltage-gated calcium channels. To confirm that calcium entry via these channels was responsible for Akt and GSK3 phosphorylation during action potential stimulation, we employed the non-selective calcium channel inhibitor flunarizine [13]. CGN cultures were stimulated with a train of 800 action potentials (80 Hz) a stimulus protocol that ensures maximal Akt and GSK3 phosphorylation [10]. As expected, a robust activity-dependent phosphorylation of both Akt (Fig. 2b) and GSK3 (Fig. 2c) was observed in the absence of flunarizine. In contrast, incubation with flunarizine (10 μM) resulted in a complete block of activity-dependent Akt and GSK3 phosphorylation (Fig. 2a–c). To ensure that this inhibition was due to blockade of calcium channels, these experiments were repeated with ionomycin stimulation, which increases [Ca^2+^]_i_ independently of calcium channel activation. Flunarizine had no effect on ionomycin-evoked Akt/GSK3 phosphorylation (Fig. 2d–f), illustrating that its inhibition of activity-dependent phosphorylation was not due to off-target effects on [Ca^2+^]_i_ levels. Thus influx of extracellular calcium via voltage-gated calcium channels is essential for activation of the Akt/GSK3 phosphorylation cascade during intense neuronal activity.

### A Localised [Ca^2+^]_i_ Increase is Essential for Akt/GSK3 Phosphorylation

**Fig. 3:**
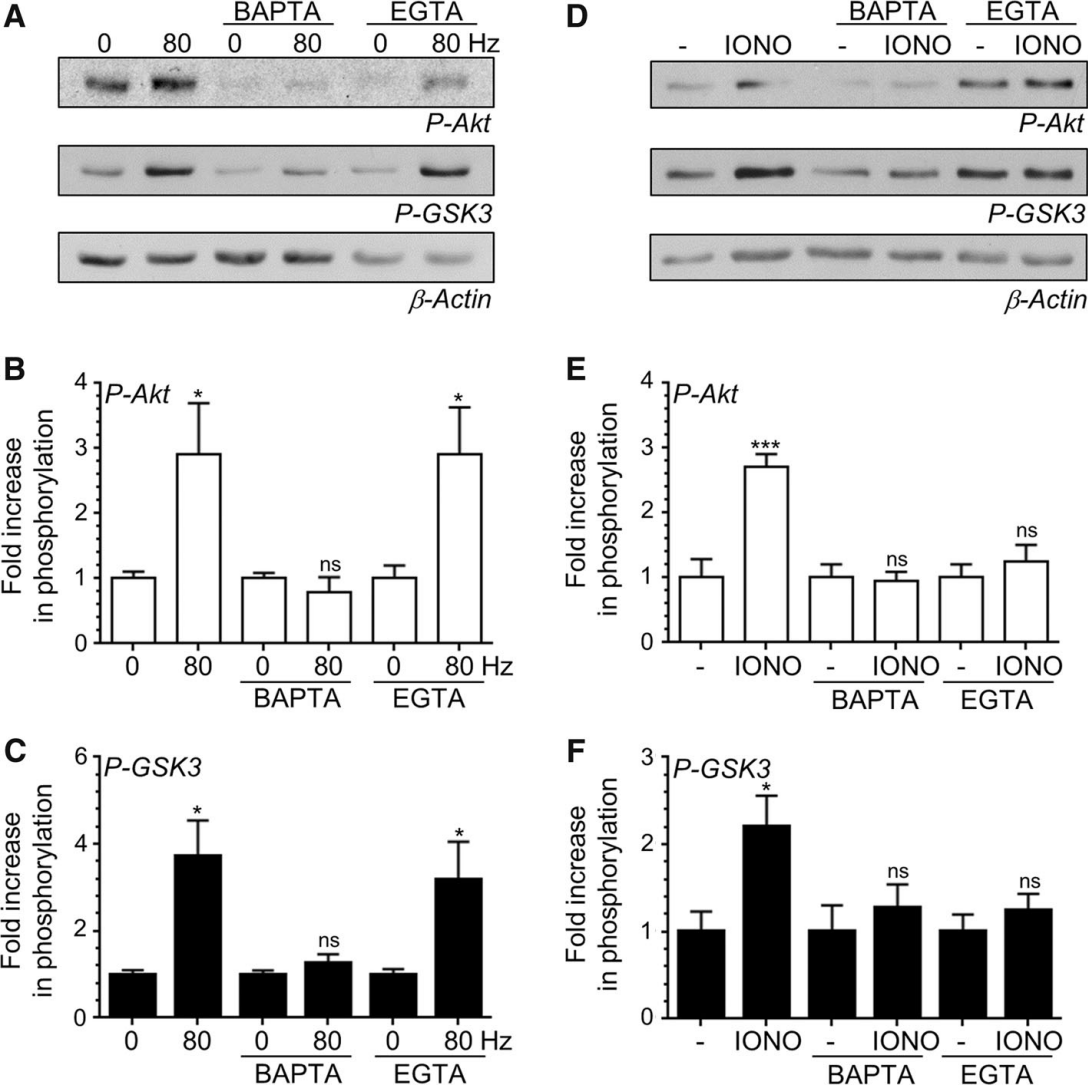
Localised Ca^2+^ influx is essential for Akt/GSK3 phosphorylation. CGNs were removed from culture medium and repolarised in incubation medium for 10 min. Cultures were then incubated with or without incubation medium supplemented with either 100 μM BAPTA-AM or EGTA-AM for 30 min. After this point CGNs were left to rest or challenged with either 800 action potentials (80 Hz) or ionomycin (IONO, 2.5 µM for 1 min). **a**, **d** Representative *blots* are displayed showing either Akt Ser473 phosphorylation (*P*-*Akt*), GSK3α/β Ser21/9 phosphorylation (*P*-*GSK3*) or β-Actin levels (*β*-*Actin*) after either action potential (**a**) or ionomycin (**d**) stimulation. **b**, **e** The fold increase in phosphorylation of Akt Ser473 (*open bars*) in response to either action potentials (**b**) or ionomycin (**e**) is displayed. **c**, **f** The fold increase in phosphorylation of GSK3α/β Ser21/9 (*closed bars*) in response to either action potentials (**c**) or ionomycin (**f**) is displayed. In all cases phosphorylation levels were corrected for protein levels using β-Actin and normalisation to the basal controls. All *error bars* represent ±SEM; n = 4 for P-Akt 80 Hz, n = 5 for P-GSK3 80 Hz, n = 7 for P-Akt IONO and n = 6 for P-GSK3 IONO (students *t* test, *ns* non-significant *p < 0.05; **p < 0.01, ***p < 0.001 basal to 80 Hz or basal to ionomycin per condition)

In central nerve terminals voltage-gated calcium channels are clustered at the active zone. During action potential stimulation the opening of these channels generates a localised microdomain of high [Ca^2+^]_i_ which is required for neurotransmitter release [14]. We next determined whether this localised microdomain is also required for Akt and GSK3 phosphorylation during intense neuronal activity by challenging CGNs with a train of 800 action potentials after prior treatment with the calcium chelators BAPTA-AM and EGTA-AM (both 100 µM). Both chelators have the same affinity for calcium but a different on-rate for binding, meaning BAPTA-AM effectively buffers [Ca^2+^]_i_ increases at the active zone, whereas EGTA-AM does not [14]. Both chelators efficiently buffer activity-dependent [Ca^2+^]_i_ increases outside the active zone. The action potential-evoked increase in both Akt and GSK3 phosphorylation was abolished in CGNs treated with BAPTA-AM, confirming the requirement for increased [Ca^2+^]_i_ (Fig. 3a–c). Interestingly, CGNs treated with EGTA-AM displayed a robust activity-dependent phosphorylation of both Akt and GSK3 (Fig. 3a–c). This suggests that the localised increase in [Ca^2+^]_i_ at the active zone during action potential stimulation is essential for the phosphorylation of Akt and GSK3. Importantly, both BAPTA-AM and EGTA-AM arrested phosphorylation of Akt and GSK3 evoked by ionomycin (Fig. 3d–f). This was expected, since ionomycin increases [Ca^2+^]_i_ globally across the nerve terminal. Thus during intense neuronal activity, a localised increase in [Ca^2+^]_i_ at the active zone is essential for triggering both Akt and GSK3 phosphorylation.

### PI3K Activity is Required for Activity-Dependent Akt and GSK3 Phosphorylation

**Fig. 4:**
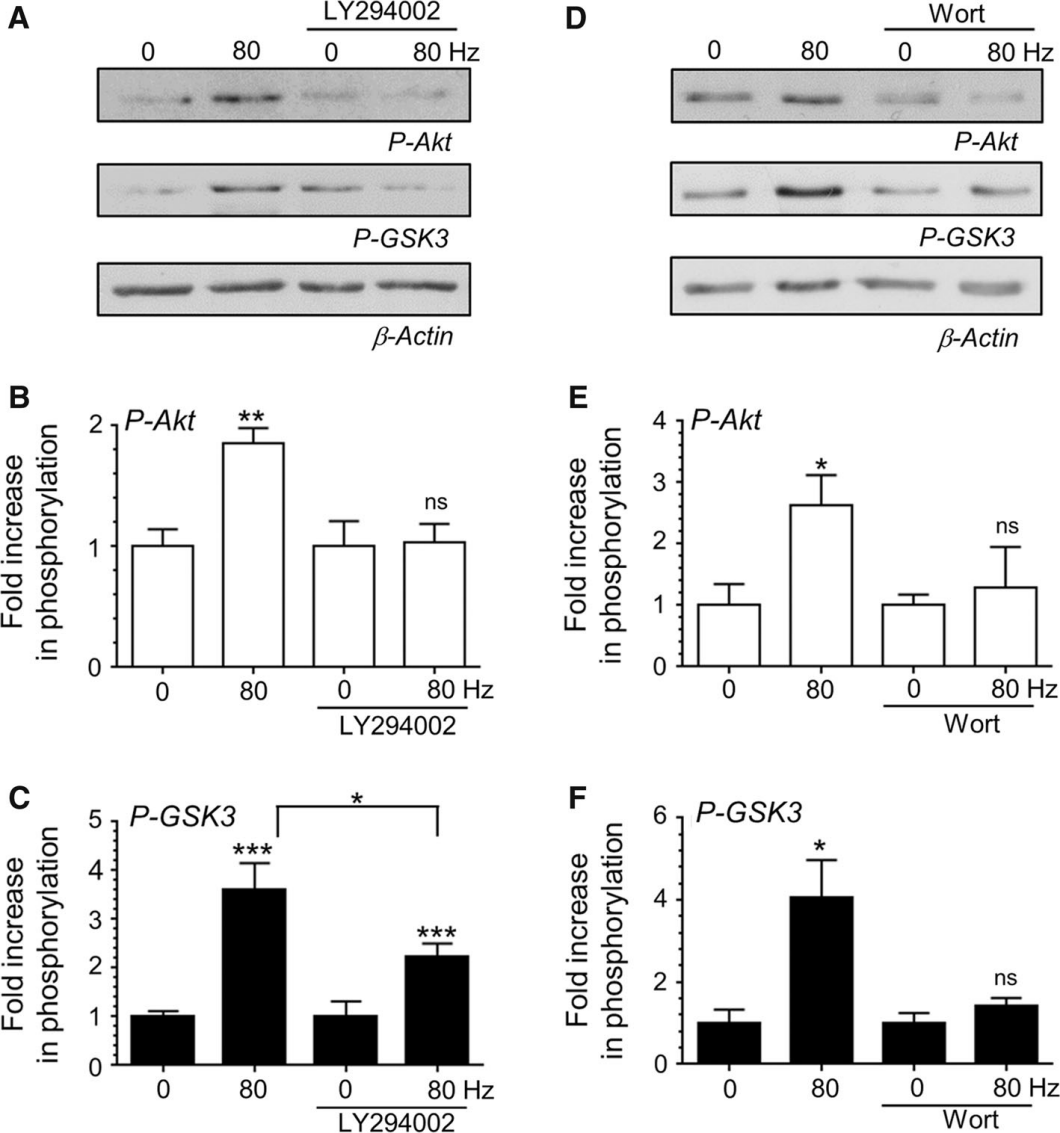
PI3K activity is essential for activity-dependent Akt/GSK3 phosphorylation. CGNs were removed from culture medium and repolarised in incubation medium for 10 min. Cultures were then incubated with or without incubation medium supplemented with either 10 μM LY294002 or 200 nM wortmannin for 10 min. After this point CGNs were left to rest or challenged with either 800 action potentials (80 Hz). **a**, **d** Representative *blots* are displayed showing either Akt Ser473 phosphorylation (*P*-*Akt*), GSK3α/β Ser21/9 phosphorylation (*P*-*GSK3*) or β-Actin levels (*β*-*Actin*) after action potential stimulation in the presence of either LY294002 (**a**) or wortmannin (**d**). **b**, **e** The fold increase in phosphorylation of Akt Ser473 (*open bars*) in the presence of either LY294002 (**b**) or wortmannin (**e**). **c**, **f** The fold increase in phosphorylation of GSK3α/β Ser21/9 (*closed bars*) in the presence of either LY294002 (**c**) or wortmannin (**f**). In all cases phosphorylation levels were corrected for protein levels using β-Actin and normalisation to the basal controls. All *error bars* represent ±SEM; LY294002—n = 6 for P-Akt, n = 16 for P-GSK3; wortmannin—n = 4 for P-Akt, n = 6 for P-GSK3 (students *t* test, *ns* non-significant, *p < 0.05; **p < 0.01; ***p < 0.001 basal to 80 Hz per condition)

To understand how activity-dependent calcium influx is coupled to downstream Akt and GSK3 phosphorylation we examined the role of PI3K. PI3K activity plays a key role in phosphorylation of Akt and subsequently GSK3 after addition of the signalling molecule BDNF to CGNs [10], however it is not known whether action potentials activate the same signalling pathway. To determine the role for PI3K in activity-dependent Akt and GSK3 phosphorylation, the PI3K inhibitors LY294002 (10 µM) and wortmannin (200 nM) were used. Inhibition of PI3K with either LY294002 or wortmannin resulted in an arrest of activity-dependent Akt phosphorylation evoked by a train of 800 action potentials (Fig. 4). In addition LY294002 and wortmannin also significantly reduced GSK3 phosphorylation (Fig. 4). Thus intense neuronal activity results in activation of PI3K, which triggers activity-dependent Akt and GSK3 phosphorylation.

**Fig. 5:**
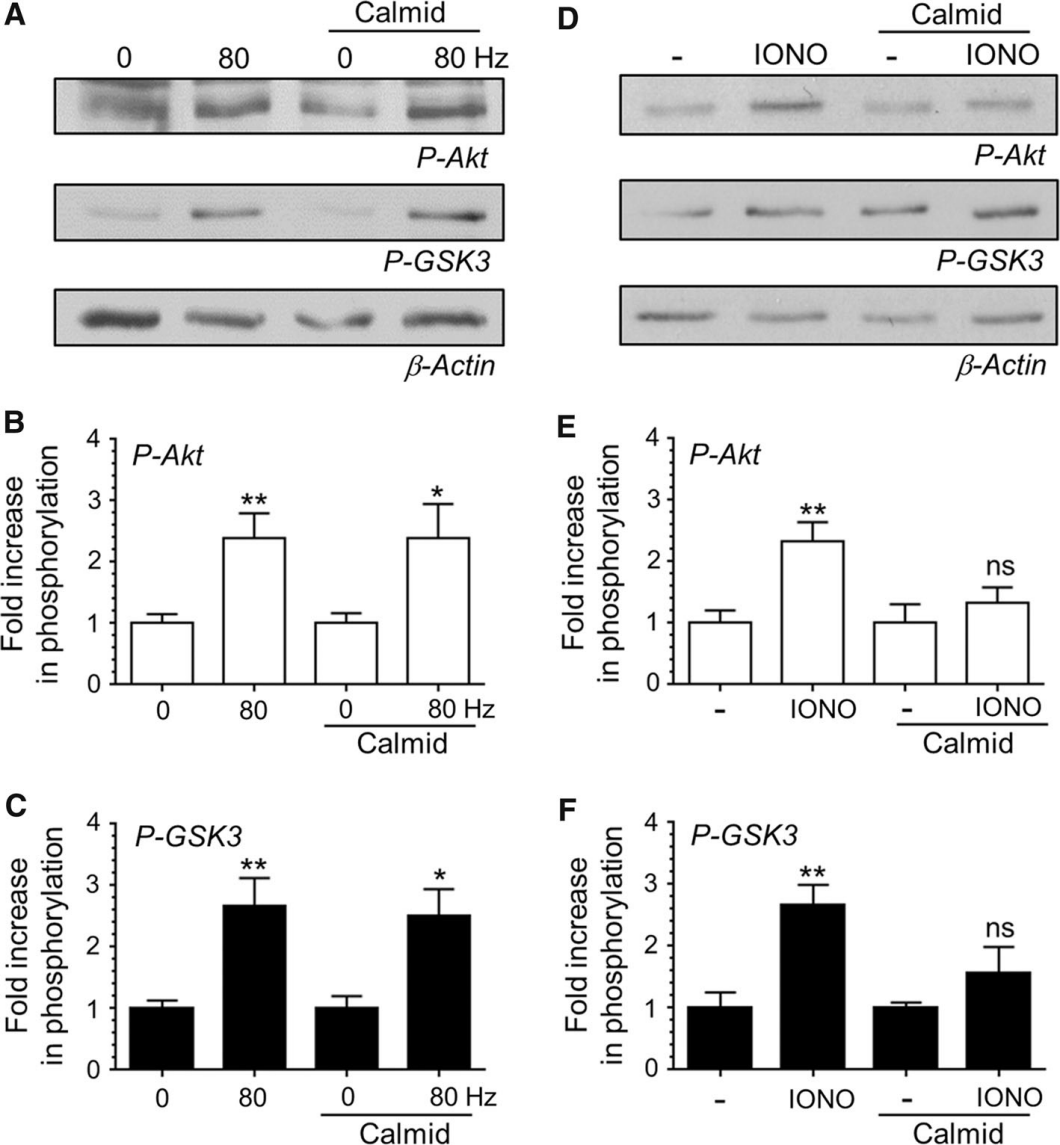
Calmodulin is not the calcium sensor for activity-dependent Akt/GSK3 phosphorylation. CGNs were removed from culture medium and repolarised in incubation medium for 10 min. Cultures were then incubated with or without incubation medium supplemented with 10 μM calmidazolium for 10 min. After this point CGNs were left to rest or challenged with either 800 action potentials (80 Hz) or ionomycin (IONO, 2.5 µM for 1 min). **a**, **d** Representative *blots* are displayed showing either Akt Ser473 phosphorylation (*P*-*Akt*), GSK3α/β Ser21/9 phosphorylation (*P*-*GSK3*) or β-Actin levels (*β*-*Actin*) after either action potential (**a**) or ionomycin (**d**) stimulation. **b**, **e** The fold increase in phosphorylation of Akt Ser473 (*open bars*) in response to either action potentials (**b**) or ionomycin (**e**) is displayed. **c**, **f** The fold increase in phosphorylation of GSK3α/β Ser21/9 (*closed bars*) in response to either action potentials (**c**) or ionomycin (**f**) is displayed. In all cases phosphorylation levels were corrected for protein levels using β-Actin and normalisation to the basal controls. All *error bars* represent ±SEM; n = 6 for P-Akt 80 Hz, n = 9 for P-GSK3 80 Hz, n = 8 for P-Akt IONO and n = 9 for P-GSK3 IONO (students *t* test, *ns* non-significant, *p < 0.05; **p < 0.01 basal to 80 Hz or basal to ionomycin per condition)

In order to transduce activity-dependent calcium influx into activation of PI3K, a calcium sensor must be present in nerve terminals, most probably at the active zone. One potential sensor is the calcium binding protein calmodulin [15]. Calmodulin can directly activate PI3K on elevation of [Ca^2+^]_i_ [16] and is thought to be required for calcineurin activation during intense neuronal activity [17]. To determine whether calmodulin can transduce increased [Ca^2+^]_i_ into PI3K activation (and downstream Akt/GSK3 phosphorylation) we used the antagonist calmidazolium. Incubation with calmidazolium (10 μM) during stimulation with 800 action potentials had no significant effect on the activity-dependent phosphorylation of either Akt or GSK3 (Fig. 5a–c). Interestingly when [Ca^2+^]_i_ was raised globally with ionomycin, calmidazolium abolished both Akt and GSK3 phosphorylation (Fig. 5d–f). Thus the activity-dependent calcium sensor for activation of the PI3K/Akt/GSK3 cascade is not calmodulin, however this cascade can be activated via calmodulin via global [Ca^2+^]_i_ increases.

## Discussion

The maintenance of neurotransmission during intense neuronal activity is reliant on the integration of activity-dependent calcium influx, multiple signalling cascades and several SV endocytosis modes [1, 2]. One such cascade is the activity-dependent inhibition of GSK3 via Akt, which is essential for the efficient dephosphorylation of dynamin I, a key event in the triggering of ADBE [10, 11]. We show here that localised calcium influx via voltage-gated channels is essential for this event to occur. Furthermore we have demonstrated a key role for PI3K, but not calmodulin, in transduction of activity-dependent calcium influx into activation of Akt.

PI3K is implicated in the regulation of secretion and membrane trafficking in a variety of systems [18–21]. One member of this family, PI3K-Class 2 α (PI3K-C2α), regulates exocytosis either indirectly via modulation of signalling pathways [22] or directly via control of the molecular fusion machinery [23, 24]. PI3K-C2α enzymatic activity is stimulated by calcium [25] and the N-terminus contains a clathrin binding site required for recruitment of the vesicle uncoating machinery [26]. This suggests that PI3K-C2α may be a good candidate involved in the transduction of activity-dependent calcium influx to the Akt signalling cascade in this work. However, PI3K-C2α is insensitive to LY294002 and wortmannin at the concentrations used in this study [27], making it unlikely that PI3K-C2α is involved in the activity-dependent activation of Akt. LY294002 and wortmannin sensitive Class I PI3Ks have been implicated at several stages of SV recycling in central nerve terminals, including replenishment of the readily releasable pool (RRP) from the reserve pool [28], generation of bulk endosomes [29] and SV generation from bulk endosomes [30]. PI3K is also required for the negative regulation of ADBE via BDNF [11]. Therefore it would be of interest to determine the activity-dependent role of PI3K in both dynamin I dephosphorylation and ADBE.

### Activity-Dependent Akt Phosphorylation at Nerve Terminals

We assessed the inhibition of GSK3 and activation of Akt respectively by monitoring their phosphorylation status. Inhibition of GSK3 occurs via phosphorylation of either Ser9 of GSK3α or Ser21 of GSK3β [9]. In this study we found that GSK3α and GSK3β behaved in an identical manner across all stimulation conditions and pharmacological manipulations. We assessed activation of Akt by determining the phosphorylation status of Ser473. Phosphorylation of both Ser473 and Thr308 are required for maximal Akt activity [31, 32] and we have previously shown that the phosphorylation status of both sites respond in a similar manner to action potential stimulation in CGNs [10]. These sites are proposed to be phosphorylated by different protein kinases however. Thr308 is a PDK1 substrate, which is activated by PI3K [31, 33], whereas Ser473 can be phosphorylated by either the mTORC2 complex [32] or DNA-PK [34]. However we show that the PI3K antagonists LY294002 and wortmannin inhibit the activity-dependent phosphorylation of Ser473. This suggests that Akt phosphorylation is mediated via PI3K at central nerve terminals and studies that attempt to model this process should take this into account.

Akt and GSK3 have a wide cellular distribution, suggesting that the increased phosphorylation we observed during action potential stimulation may not be occurring at nerve terminals. We contend that these events are presynaptic, since identical stimulation protocols and modulation of both Akt and GSK3 activity-dependent phosphorylation directly impact on the phosphorylation status of dynamin I and ADBE [10, 11]. Since both dynamin I expression and ADBE is restricted to nerve terminals [2, 35] we argue that the activity-dependent changes in Akt and GSK3 phosphorylation are presynaptic. This statement is almost certainly not true for ionomycin-evoked changes, since this ionophore will raise [Ca^2+^]_i_ uniformly throughout the neuron. Ionomycin is used throughout this study as a positive control to confirm the specificity of both flunarizine and EGTA-AM in inhibiting calcium channels and global calcium increases respectively. The inhibition of ionomycin-evoked Akt and GSK3 phosphorylation by calmidazolum is intriguing however and may reflect altered activity of these enzymes by postsynaptic calcium, where Akt and GSK3 are known to have postsynaptic functions [36, 37].

A number of studies have investigated the relationship between both neuronal activity and calcium influx in the activation of Akt. Previous work in CGNs showed that chronic hyperkalemic stimulation resulted in increased Akt phosphorylation, however this was insensitive to PI3K antagonists [38]. This most likely reflects postsynaptic activation of Akt, since permanent depolarisation will reverse electrogenic glutamate transporters on the plasma membrane [39] raising extracellular glutamate concentrations and thus activating postsynaptic glutamate receptors. In agreement, shorter periods of stimulation with elevated KCl resulted in increased Akt phosphorylation which was reversed by PI3K antagonists [40].

### Transduction of Calcium Influx into Akt Phosphorylation

We have shown that intense neuronal activity causes Akt phosphorylation via localised calcium microdomains. This suggests that a sensor must be located within the vicinity of this microdomain to couple calcium influx to PI3K/Akt activation. We have shown that calmodulin is unlikely to be the sensor since the antagonist calmidazolium was unable to inhibit action potential-evoked Akt phosphorylation. Furthermore the ability of calmidazolium to arrest ionomycin-evoked Akt phosphorylation is in agreement with the proposed location of calmodulin in the wider cytosol [15].

How could activity-dependent calcium influx be coupled to Akt phosphorylation? One potential candidate for the activity-dependent calcium sensor is the family of Ras-GRFs [41]. Ras-GRF activates the small G-protein Ras in a calcium-dependent manner [42] and overexpression of dominant negative Ras inhibits Akt phosphorylation evoked by KCl depolarisation in peripheral neurons [40]. Importantly Ras-GRF2 knockout mice display altered paired pulse facilitation, suggesting it is localised in the correct location to be activated by presynaptic calcium influx [43]. However the mechanism via which Ras-GRF is activated by calcium influx is thought to be via calcium-bound calmodulin binding to its IQ domain [42, 44]. Since calmodulin inhibition had no effect on activity-dependent Akt phosphorylation, it may be that Ras-GRF is not the sensor for this event.

A second possibility is that there may not be calcium a sensor at all. In this model the requirement for localised calcium increases is an indirect result of the presynaptic release of growth factors such as BDNF. This event only occurs in neurons during intense neuronal activity, very similar to that observed in this study [45]. This is potentially important for Akt phosphorylation, since PI3K can activate either PDK1 or the mTORC2 complex via growth factor receptors [46]. In support, we have shown that exogenous addition of BDNF triggers phosphorylation of Ser473 in CGNs via a potential PI3K-dependent route [11]. Thus our manipulations to either block calcium entry or buffer the calcium microdomain should also arrest BDNF release during intense activity, stopping it feeding back to increase Akt phosphorylation via PI3K. This hypothesis is testable using agents that sequester extracellular BDNF and we are currently investigating the effect of these agents on activity-dependent Akt phosphorylation [47].

In summary, we have shown that during intense neuronal activity phosphorylation of Akt (and subsequent inhibition of GSK3) is dependent on localised calcium microdomains formed by voltage-gated calcium channels. The identity of the sensor or release mechanism which transduce increased [Ca^2+^]_i_ remains unidentified. This is an important question to address, since we predict that modulation of these events should impact on the extent of ADBE in central nerve terminals.

## References

[1] Kononenko NL, Haucke V (2015). Molecular mechanisms of presynaptic membrane retrieval and synaptic vesicle reformation. Neuron.

[2] Clayton EL, Cousin MA (2009). The molecular physiology of activity-dependent bulk endocytosis of synaptic vesicles. J Neurochem.

[3] Granseth B, Odermatt B, Royle SJ, Lagnado L (2006). Clathrin-mediated endocytosis is the dominant mechanism of vesicle retrieval at hippocampal synapses. Neuron.

[4] Watanabe S, Rost BR, Camacho-Perez M, Davis MW, Sohl-Kielczynski B, Rosenmund C, Jorgensen EM (2013). Ultrafast endocytosis at mouse hippocampal synapses. Nature.

[5] Watanabe S, Trimbuch T, Camacho-Perez M, Rost BR, Brokowski B, Sohl-Kielczynski B, Felies A, Davis MW, Rosenmund C, Jorgensen EM (2014). Clathrin regenerates synaptic vesicles from endosomes. Nature.

[6] Kokotos AC, Cousin MA (2014). Synaptic vesicle generation from central nerve terminal endosomes. Traffic.

[7] Clayton EL, Anggono V, Smillie KJ, Chau N, Robinson PJ, Cousin MA (2009). The phospho-dependent dynamin-syndapin interaction triggers activity-dependent bulk endocytosis of synaptic vesicles. J Neurosci.

[8] Clayton EL, Sue N, Smillie KJ, O’Leary T, Bache N, Cheung G, Cole AR, Wyllie DJ, Sutherland C, Robinson PJ, Cousin MA (2010). Dynamin I phosphorylation by GSK3 controls activity-dependent bulk endocytosis of synaptic vesicles. Nat Neurosci.

[9] Frame S, Cohen P (2001). GSK3 takes centre stage more than 20 years after its discovery. Biochem J.

[10] Smillie KJ, Cousin MA (2012). Akt/PKB controls the activity-dependent bulk endocytosis of synaptic vesicles. Traffic.

[11] Smillie KJ, Pawson J, Perkins EM, Jackson M, Cousin MA (2013). Control of synaptic vesicle endocytosis by an extracellular signalling molecule. Nat Commun.

[12] Tan TC, Valova VA, Malladi CS, Graham ME, Berven LA, Jupp OJ, Hansra G, McClure SJ, Sarcevic B, Boadle RA, Larsen MR, Cousin MA, Robinson PJ (2003). Cdk5 is essential for synaptic vesicle endocytosis. Nat Cell Biol.

[13] Cousin MA, Nicholls DG, Pocock JM (1993). Flunarizine inhibits both calcium-dependent and -independent release of glutamate from synaptosomes and cultured neurones. Brain Res.

[14] Adler EM, Augustine GJ, Duffy SN, Charlton MP (1991). Alien intracellular calcium chelators attenuate neurotransmitter release at the squid giant synapse. J Neurosci.

[15] Chin D, Means AR (2000). Calmodulin: a prototypical calcium sensor. Trends Cell Biol.

[16] Joyal JL, Burks DJ, Pons S, Matter WF, Vlahos CJ, White MF, Sacks DB (1997). Calmodulin activates phosphatidylinositol 3-kinase. J Biol Chem.

[17] Wu XS, McNeil BD, Xu J, Fan J, Xue L, Melicoff E, Adachi R, Bai L, Wu LG (2009). Ca(2+) and calmodulin initiate all forms of endocytosis during depolarization at a nerve terminal. Nat Neurosci.

[18] Balla T (2013). Phosphoinositides: tiny lipids with giant impact on cell regulation. Physiol Rev.

[19] Chasserot-Golaz S, Coorssen JR, Meunier FA, Vitale N (2010). Lipid dynamics in exocytosis. Cell Mol Neurobiol.

[20] Osborne SL, Wen PJ, Meunier FA (2006). Phosphoinositide regulation of neuroexocytosis: adding to the complexity. J Neurochem.

[21] Mazza S, Maffucci T (2011). Class II phosphoinositide 3-kinase C2alpha: what we learned so far. Int J Biochem Mol Biol.

[22] Leibiger B, Moede T, Uhles S, Barker CJ, Creveaux M, Domin J, Berggren PO, Leibiger IB (2010). Insulin-feedback via PI3K-C2alpha activated PKBalpha/Akt1 is required for glucose-stimulated insulin secretion. FASEB J.

[23] Meunier FA, Osborne SL, Hammond GR, Cooke FT, Parker PJ, Domin J, Schiavo G (2005). Phosphatidylinositol 3-kinase C2alpha is essential for ATP-dependent priming of neurosecretory granule exocytosis. Mol Biol Cell.

[24] Dominguez V, Raimondi C, Somanath S, Bugliani M, Loder MK, Edling CE, Divecha N, da Silva-Xavier G, Marselli L, Persaud SJ, Turner MD, Rutter GA, Marchetti P, Falasca M, Maffucci T (2011). Class II phosphoinositide 3-kinase regulates exocytosis of insulin granules in pancreatic beta cells. J Biol Chem.

[25] Wen PJ, Osborne SL, Morrow IC, Parton RG, Domin J, Meunier FA (2008). Ca^2+^-regulated pool of phosphatidylinositol-3-phosphate produced by phosphatidylinositol 3-kinase C2alpha on neurosecretory vesicles. Mol Biol Cell.

[26] Gaidarov I, Smith ME, Domin J, Keen JH (2001). The class II phosphoinositide 3-kinase C2alpha is activated by clathrin and regulates clathrin-mediated membrane trafficking. Mol Cell.

[27] Domin J, Pages F, Volinia S, Rittenhouse SE, Zvelebil MJ, Stein RC, Waterfield MD (1997). Cloning of a human phosphoinositide 3-kinase with a C2 domain that displays reduced sensitivity to the inhibitor wortmannin. Biochem J.

[28] Cousin MA, Malladi CS, Tan TC, Raymond CR, Smillie KJ, Robinson PJ (2003). Synapsin I-associated phosphatidylinositol 3-kinase mediates synaptic vesicle delivery to the readily releasable pool. J Biol Chem.

[29] Holt M, Cooke A, Wu MM, Lagnado L (2003). Bulk membrane retrieval in the synaptic terminal of retinal bipolar cells. J Neurosci.

[30] Richards DA, Rizzoli SO, Betz WJ (2004). Effects of wortmannin and latrunculin A on slow endocytosis at the frog neuromuscular junction. J Physiol.

[31] Alessi DR, James SR, Downes CP, Holmes AB, Gaffney PR, Reese CB, Cohen P (1997). Characterization of a 3-phosphoinositide-dependent protein kinase which phosphorylates and activates protein kinase Balpha. Curr Biol.

[32] Sarbassov DD, Guertin DA, Ali SM, Sabatini DM (2005). Phosphorylation and regulation of Akt/PKB by the rictor-mTOR complex. Science.

[33] Brazil DP, Hemmings BA (2001). Ten years of protein kinase B signalling: a hard Akt to follow. Trends Biochem Sci.

[34] Bozulic L, Hemmings BA (2009). PIKKing on PKB: regulation of PKB activity by phosphorylation. Curr Opin Cell Biol.

[35] Ferguson SM, De Camilli P (2012). Dynamin, a membrane-remodelling GTPase. Nat Rev Mol Cell Biol.

[36] Li Z, Jo J, Jia JM, Lo SC, Whitcomb DJ, Jiao S, Cho K, Sheng M (2010). Caspase-3 activation via mitochondria is required for long-term depression and AMPA receptor internalization. Cell.

[37] Peineau S, Taghibiglou C, Bradley C, Wong TP, Liu L, Lu J, Lo E, Wu D, Saule E, Bouschet T, Matthews P, Isaac JT, Bortolotto ZA, Wang YT, Collingridge GL (2007). LTP inhibits LTD in the hippocampus via regulation of GSK3beta. Neuron.

[38] Lafon-Cazal M, Perez V, Bockaert J, Marin P (2002). Akt mediates the anti-apoptotic effect of NMDA but not that induced by potassium depolarization in cultured cerebellar granule cells. Eur J Neurosci.

[39] Nicholls DG (1993). The glutamatergic nerve terminal. Eur J Biochem/FEBS.

[40] Vaillant AR, Mazzoni I, Tudan C, Boudreau M, Kaplan DR, Miller FD (1999). Depolarization and neurotrophins converge on the phosphatidylinositol 3-kinase-Akt pathway to synergistically regulate neuronal survival. J Cell Biol.

[41] Fernandez-Medarde A, Santos E (2011). The RasGrf family of mammalian guanine nucleotide exchange factors. Biochim Biophys Acta.

[42] Farnsworth CL, Freshney NW, Rosen LB, Ghosh A, Greenberg ME, Feig LA (1995). Calcium activation of Ras mediated by neuronal exchange factor Ras-GRF. Nature.

[43] Li S, Tian X, Hartley DM, Feig LA (2006). Distinct roles for Ras-guanine nucleotide-releasing factor 1 (Ras-GRF1) and Ras-GRF2 in the induction of long-term potentiation and long-term depression. J Neurosci.

[44] Bahler M, Rhoads A (2002). Calmodulin signaling via the IQ motif. FEBS Lett.

[45] Matsuda N, Lu H, Fukata Y, Noritake J, Gao H, Mukherjee S, Nemoto T, Fukata M, Poo MM (2009). Differential activity-dependent secretion of brain-derived neurotrophic factor from axon and dendrite. J Neurosci.

[46] Oh WJ, Jacinto E (2011). mTOR complex 2 signaling and functions. Cell Cycle.

[47] Chen DY, Bambah-Mukku D, Pollonini G, Alberini CM (2012). Glucocorticoid receptors recruit the CaMKIIalpha-BDNF-CREB pathways to mediate memory consolidation. Nat Neurosci.

